# A Localized Scalable DNA Logic Circuit System Based on the DNA Origami Surface

**DOI:** 10.3390/ijms26052043

**Published:** 2025-02-26

**Authors:** Zhen Tang, Shiyin Li, Chunlin Chen, Zhaohua Zhou, Zhixiang Yin

**Affiliations:** 1 School of Mathematics, Physics and Statistics, Shanghai University of Engineering Science, Shanghai 201620, China; ztang1994@sues.edu.cn (Z.T.); vimoly@126.com (S.L.); chenchulin1119@163.com (C.C.); curezhaohua@163.com (Z.Z.); 2 Institute for Frontier Medical Technology, Shanghai Frontiers Science Research Center for Druggability of Cardiovascular Noncoding RNA, Shanghai University of Engineering Science, Shanghai 201620, China; 3 Center of Intelligent Computing and Applied Statistics, Shanghai University of Engineering Science, Shanghai 201620, China

**Keywords:** DNA computing, DNA logic circuits, strand displacement reaction, DNA origami

## Abstract

DNA (Deoxyribonucleic Acid) logic circuit systems provide a powerful arithmetic architecture for the development of molecular computations. DNA nanotechnology, particularly DNA origami, provides a nanoscale addressable surface for DNA logic circuit systems. Although molecular computations based on DNA origami surfaces have received significant attention in research, there are still obstacles to constructing localized scalable DNA logic circuit systems. Here, we developed elementary DNA logic circuits on a DNA origami surface by employing the strand displacement reaction (SDR) to realize the localized scalable DNA logic circuit systems. We showed that the constructed elementary logic circuits can be scaled up to the localized DNA logic circuit systems that perform arbitrary digital computing tasks, including square root functions, full adder and full subtractor. We used a 50% reduction in the number of localized DNA logic components, compared to localized logic systems based on the threshold strategy. We further demonstrated that the localized DNA logic circuit systems for three-satisfiability (3-SAT) problem solving and disease classification can be implemented using the constructed elementary DNA logic circuits. We expect our approach to provide a new design paradigm for the development of molecular computations and their applications in complex mathematical problem solving and disease diagnosis.

## 1. Introduction

DNA (Deoxyribonucleic Acid) computing has an excellent ability to process information in parallel, benefiting from liquid-phase biomolecular interactions and the highly programmable nature of DNA molecules [1,2,3]. A seminal study in this field is that of Adleman, who solved the seven-city Hamilton path problem using DNA coding in 1994 [4]. DNA molecules have been used as computing substrates for computation and information processing and have evolved from the silicon-based computing paradigm to the parallel biocomputing paradigm. In DNA computing, DNA logic circuits are essential arithmetic architectures for digital computing, neural networks, molecular machines and algorithmic decision-making [5,6,7,8]. Concurrently, the excellent biocompatibility and nanoscale size of DNA molecules have facilitated the application of DNA logic circuits in the fields of biosensors, disease diagnosis and intelligent drug delivery [9,10,11,12]. Overall, DNA logic circuit systems have emerged as a quality interface for the cross-fusion of biotechnology and information technology.

Over the past few decades, the strand displacement reaction (SDR) has been frequently employed as a reaction protocol to implement logical operations and information interactions in a DNA logic circuit system due to it being enzyme-free, programmable and high-efficiency [13,14,15,16,17,18]. In a DNA logic circuit system, single-stranded DNA is commonly used to represent the input of the binary digits 0/1, random collisions occur between DNA logic elements based on Brownian motion, the interactions between DNA molecules are triggered based on SDR protocol, and the logic outputs are read through signals such as fluorescence. However, the operation of discrete DNA logic systems in a solution is entirely dependent on the interactions between the discrete DNA logic components mediated by complex sequences. Such factors cause scalable discrete DNA logic circuit systems to suffer from slow dynamics and non-reusable sequences.

To counter such issues, researchers have begun to introduce spatial constraints to construct fast and modular spatially localized DNA logic circuit systems. Several theoretical studies on the construction of spatially localized DNA circuit systems have been proposed [19,20,21]. With the development of DNA nanotechnology, particularly DNA origami, new design concepts for localized DNA circuit systems have been established [22,23,24,25,26,27]. DNA origami involves folding a long DNA scaffold strand into a two-dimensional rectangular platform. These DNA origami platforms provide an ideal surface for the construction of localized DNA logic systems, as the platforms are accompanied by uniformly distributed addressable sites and spatial constraints. In recent studies, several representative works have been proposed, such as a cargo sorting robot based on a two-dimensional DNA origami platform, single-molecule DNA navigators capable of performing maze searches, a spatially localized modular DNA circuit architecture based on the threshold strategy, a spatially localized DNA linear classifier for cancer diagnosis, and a strategy for high-speed sequential DNA computing using a solid-state DNA origami register [28,29,30,31,32].

Despite these advances, constructing localized scalable DNA logic circuit systems remains a challenge. Here, we first constructed elementary two-input AND and OR logic circuits on a DNA origami surface, where logic operations were realized by triggering the SDR. We then showed that the constructed elementary logic circuits can be scaled up to form localized DNA logic circuit systems that perform arbitrary digital computing tasks, including square root functions, full adder and full subtractor. Lastly, we programmed and designed the elementary logic circuits and realized the application of the localized DNA logic circuit systems in three-satisfiability (3-SAT) problem solving and disease classification. In this work, we employed Visual DSD software (version “v2015-0325”) to simulate all the constructed DNA logic circuit systems, and the results showed that all the systems exhibit good stability and feasibility.

## 2. Results

### 2.1. Construction and Implementation of Elementary Logic Circuits and Cascades on the DNA Origami Surface

**Figure 1:**
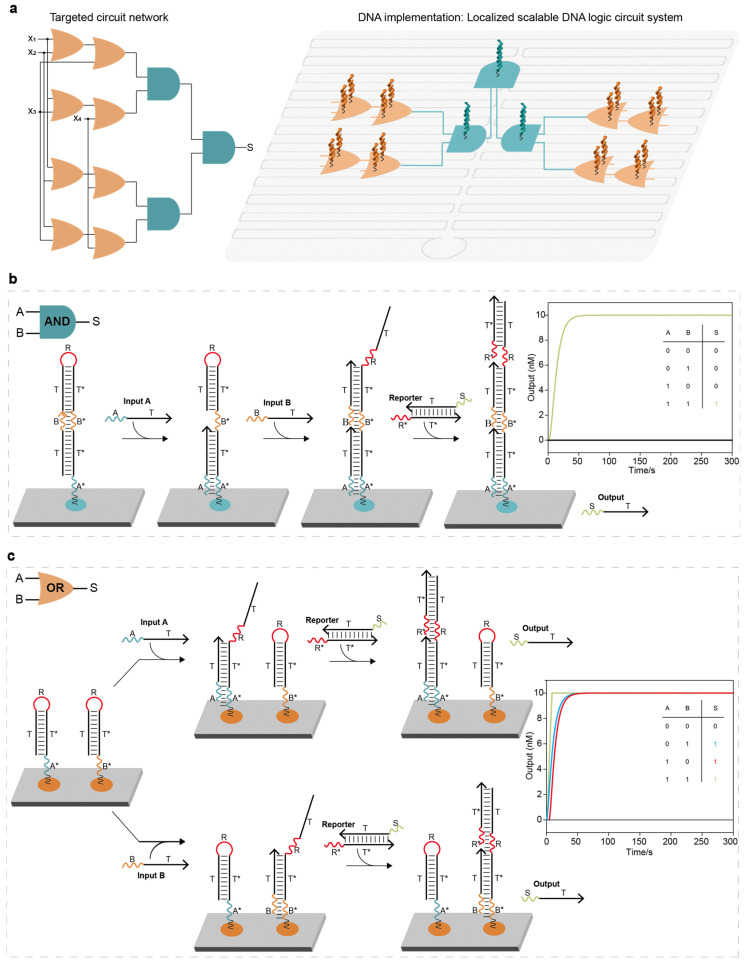
Construction of the localized elementary DNA logic circuit: (**a**) An abstraction of a localized DNA logic circuit system. Scaling up elementary logic circuits into localized DNA logic circuit systems through deliberate programming allows for the realization of a targeted circuit network. (**b**) Elementary two-input AND logic circuit. The entire system consisted of a localized DNA complex (with two toeholds A* and B*) and a reporter molecule. The base sequences of domain and domain* of the same letter were completely complementary. When two signal strands were added, the hairpin of the DNA complex was opened and reacted with the reporter molecule, displacing the output strand and producing a high signal. (**c**) Elementary two-input OR logic circuit. The entire system consisted of two localized hairpin DNA strands with two toeholds (A* and B*) and a reporter molecule. Once an input strand was added, the hairpin of the DNA strand was opened and reacted with the reporter molecule, displacing the output strand and producing a high signal. The colorful lines corresponded to reaction kinetics curves with the same color numbers.

In localized scalable DNA logic circuit systems, elementary logic circuits can be programmed and cascaded as the smallest logic units on the DNA origami surface to achieve the desired function (Figure 1a). One reported approach to designing elementary logic circuits on the DNA origami surface is the “threshold strategy”, which involves the use of a threshold DNA strand to compete with the output DNA strand to block signal propagation [29]. The toehold of the output DNA strand (3 nt (nucleotide)) was designed to be a truncated version of the threshold DNA strand (6 nt), which resulted in the signal preferentially binding to the threshold DNA strand until two signals were present. However, the “threshold strategy” requires four DNA strands to be anchored on the DNA origami surface to implement the elementary AND logic circuits (the anchoring efficiency of circuit components on the DNA origami surface impacts the performance of localized DNA logic circuit systems, Supplementary Text S1.1, Table S1 and Figure S1) and purposefully reduces the speed of signal propagation (the difference in signal propagation speed can cause the input strand to preferentially bind to the threshold hairpin strand, resulting in signal blocking). To more succinctly implement an elementary two-input AND logic circuit, we used only a DNA complex formed by base-pairing a hairpin DNA with a single strand of DNA (Figure 1b and Supplementary Figure S2). The DNA complex possessed two toeholds (A* and B*) that bind specifically to the input strands and that are confined to the origami surface by extending the poly 5T linker through staples within the DNA origami. When at most one input strand was added, the hairpin of the DNA complex could not be opened and could not react with the reporter molecule, resulting in a low output signal. When the two input strands were added, signal strand A first bound to toehold A*, and toehold B* was exposed with the SDR. Signal strand B bound to toehold B*, the hairpin of the DNA complex was turned on, and the exposed toehold R bound to the toehold R* of the reporter molecule, resulting in a high output signal (Supplementary Figure S3a). To implement an elementary two-input OR logic circuit, we used two simple hairpin DNA strands with two toeholds (A* and B*) that bind specifically to the input strands (Figure 1c). Once an input strand was added, the hairpin of the DNA strand was opened, and the exposed toehold R bound to the toehold R* of the reporter molecule, resulting in a high output signal (Supplementary Figure S3b).

The elementary two-input AND logic circuit system based on the threshold strategy had seven circuit components, four of which were anchored on the DNA origami surface (Supplementary Figure S4a). The constructed elementary two-input AND logic circuit system had two components (a nearly 71% reduction of the circuit components, Supplementary Figure S4b,e), with only one component anchored to the DNA origami surface (nearly 75% reduction, Supplementary Figure S4f). Compared to the threshold strategy, the constructed elementary two-input OR logic circuit had a total of three components (a nearly 40% reduction, Supplementary Figure S4c–e), of which only two components are anchored to the DNA origami surface (a nearly 33% reduction, Supplementary Figure S4f).

**Figure 2:**
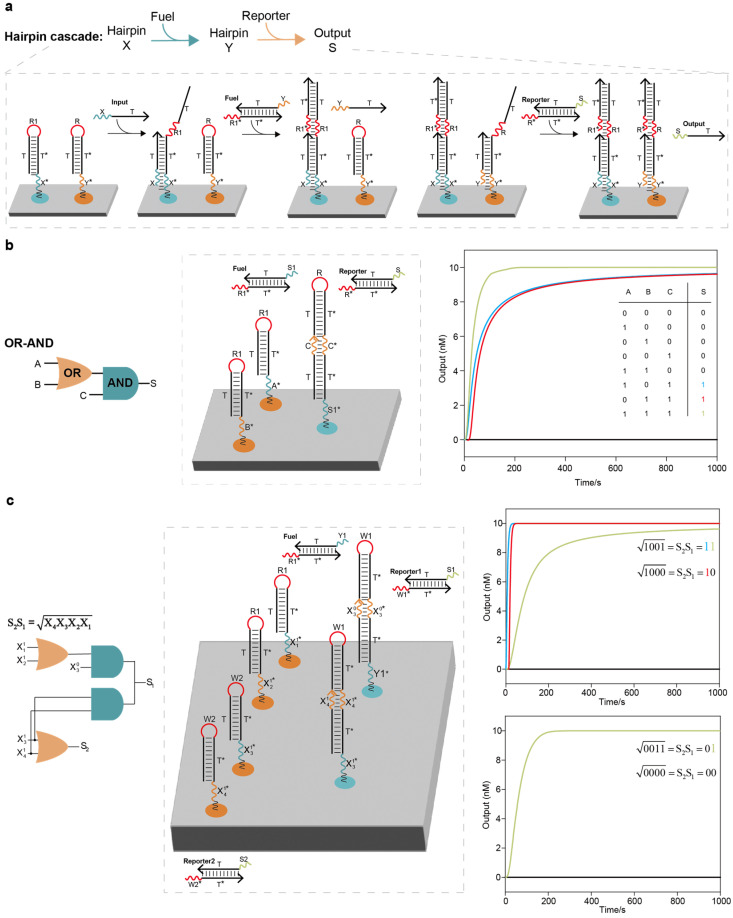
Implementation of the localized elementary DNA logic circuit cascade and localized DNA logic circuit system for computing the floor of the square root of a four-bit binary number: (**a**) Hairpin cascade. The two hairpin DNA strands were cascaded with the assistance of the fuel strand. (**b**) OR–AND circuit cascade. The entire system consisted of 1 elementary OR circuit, 1 AND circuit, 1 fuel strand and 1 reporter molecule. For the localized OR–AND logic circuit system based on the threshold strategy, it was necessary to anchor roughly 6 circuit components on the DNA origami surface; in our work, however, it was only necessary to anchor 3 circuit components, reducing the required number of strands by nearly 50%. (**c**) Localized DNA logic circuit system for computing the floor of the square root of a four-bit binary number. The entire circuit system had two layers, including the cascade of 2 OR circuits and 2 AND circuits, 1 fuel strand and 2 reporter molecules. For the circuit system based on the threshold strategy to compute the floor of the square root of a four-bit binary number, it was necessary to anchor roughly 12 circuit components on the DNA origami surface; in our work, however, it was only necessary to anchor 6 components, reducing the required number of strands by nearly 50%. Four representative examples are shown in the right column.

The cascadability of DNA logic circuits is a prerequisite for elementary logic circuits to be combined into large-scale DNA logic circuit systems. We first showed how two hairpin DNA strands cascade and interact on the DNA origami surface. As shown in Figure 2a, the fuel strand was designed to assist in the information interaction between the two hairpin DNA strands. When an input strand was added, it reacted with the first hairpin strand, and the exposed toehold R1 bound specifically to the toehold R1* of the fuel strand. The displaced single-strand DNA reacted with the second hairpin, and the exposed toehold R bound specifically to the toehold R* of the reporter molecule, resulting in a high output signal and completing the cascade interaction between the two hairpins. We demonstrated a localized OR–AND logic circuit system consisting of one elementary OR circuit, one AND circuit, one fuel strand and one reporter molecule (Figure 2b). For the localized OR–AND logic circuit system based on the threshold strategy, it was necessary to anchor roughly six circuit components on the DNA origami surface; in our work, however, it was only necessary to anchor three circuit components, reducing the required number of strands by nearly 50% (Supplementary Figure S5a,c,e). We tested all input combinations using Visual DSD software, with the outputs moving to the correct signals. The simulation results showed that all the reactions were completed in a short time span, and the constructed elementary logic circuits can effectively cascade and interact.

### 2.2. Scaling up the Localized DNA Logic Circuit Systems for Digital Computing

To exemplify that scaling up elementary DNA logic circuits can realize arbitrary digital computation tasks, we demonstrated a circuit system that computes the floor of the square root of a four-bit binary number, a full adder circuit system and a full subtractor system. DNA logic circuit systems may produce a false output before all inputs have been added, as a result of the NOT gate having already generated a signal without inputs. Therefore, dual-rail logic is necessary in scalable DNA logic circuit systems. Dual-rail logic refers to the fact that each input X in a logic gate is replaced by a pair of inputs, X1 and X0, representing the binary numbers “1” and “0”, respectively. The layout of the localized circuit system for computing the floor of the square root of a four-bit binary number on the DNA origami surface is shown in Figure 2c. The entire circuit system had two layers, including the cascade of two OR circuits and two AND circuits, one fuel strand and two reporter molecules. For the circuit system based on the threshold strategy to compute the floor of the square root of a four-bit binary number, it was necessary to anchor roughly 12 circuit components on the DNA origami surface; in our work, however, it was only necessary to anchor 6 components, reducing the number of required strands by nearly 50% (Supplementary Figure S5b,d,f). We tested all 16 possible four-bit inputs from 0000 to 1111 using Visual DSD software, and all the outputs moved to the correct signals (Supplementary Figure S6). Four representative examples are shown in the right column of Figure 2c.

**Figure 3:**
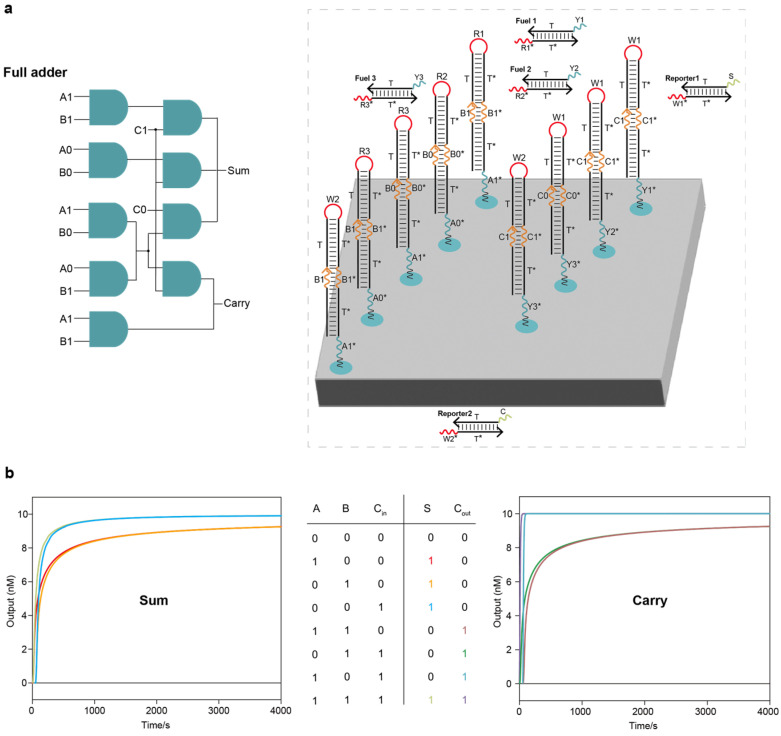
Localized DNA full adder circuit system: (**a**) The layout of the DNA full adder circuit system on the DNA origami surface. The entire full adder circuit system had two layers, including 9 AND circuits, 3 fuel strands and 2 reporter molecules. (**b**) Test results under all input combinations using Visual DSD software.

**Figure 4:**
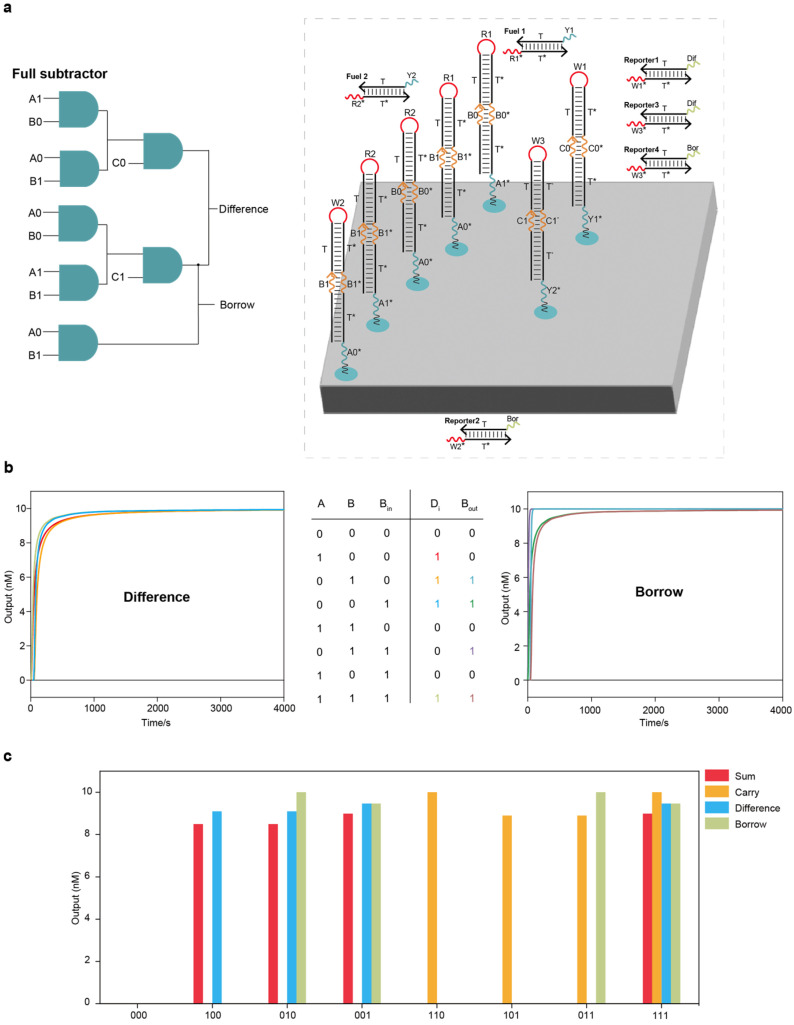
Localized DNA full subtractor circuit system and parallel computing of full adder and full subtractor circuit systems: (**a**) The layout of the DNA full subtractor circuit system on the DNA origami surface. The entire full subtractor circuit system had two layers, including 7 AND circuits, 2 fuel strands and 4 reporter molecules. (**b**) Test results under all input combinations using Visual DSD software. (**c**) Test results of parallel computing of full adder and full subtractor circuit systems using Visual DSD software.

We further scaled up the circuit systems to implement the addition and subtraction of binary numbers. A full adder is a circuit system that implements binary addition operations with an additional Carry input, C_in_, as the third input, producing two outputs, a sum (S) and a Carry (C_out_). The layout of the DNA full adder circuit system on the DNA origami surface is shown in Figure 3a. The entire full adder circuit system had two layers, including nine AND circuits, three fuel strands and two reporter molecules. We tested all input combinations using Visual DSD software, and all the outputs went to the correct signals (Figure 3b). A full subtractor is a circuit system that implements the binary subtraction operations with a borrowed input from the lower bit, Bin, as the third input, producing two outputs, a difference (D_i_) and a borrowed output to the higher bit (B_out_). The layout of the DNA full subtractor circuit system on the DNA origami surface is shown in Figure 4a. The entire full subtractor circuit system had two layers, including seven AND circuits, two fuel strands and four reporter molecules. We tested all input combinations using Visual DSD software, and all the outputs moved to the correct signals (Figure 4b).

Notably, we attempted to place the full adder and full subtractor circuit systems on the same DNA origami surface (Supplementary Figure S7a). The placement of the full adder and full subtractor on the same DNA origami surface can realize parallel computation because of the high parallelism of the biochemical reaction; put simply, the same set of binary numbers can be added and subtracted simultaneously (Figure 4c and Supplementary Figure S7b,c). Here, DNA origami can be similarly viewed as a liquid-phase biochip, and the DNA strands anchored to the DNA origami surface are analogous to circuit components on a semiconductor chip. Unlike semiconductor chips, DNA-based biochips have high parallelism, and this change from the underlying computing mode provides DNA computing with strong computing power.

### 2.3. Integrated Localized DNA Logic Circuit Systems for Solving the 3-SAT Problem

**Figure 5:**
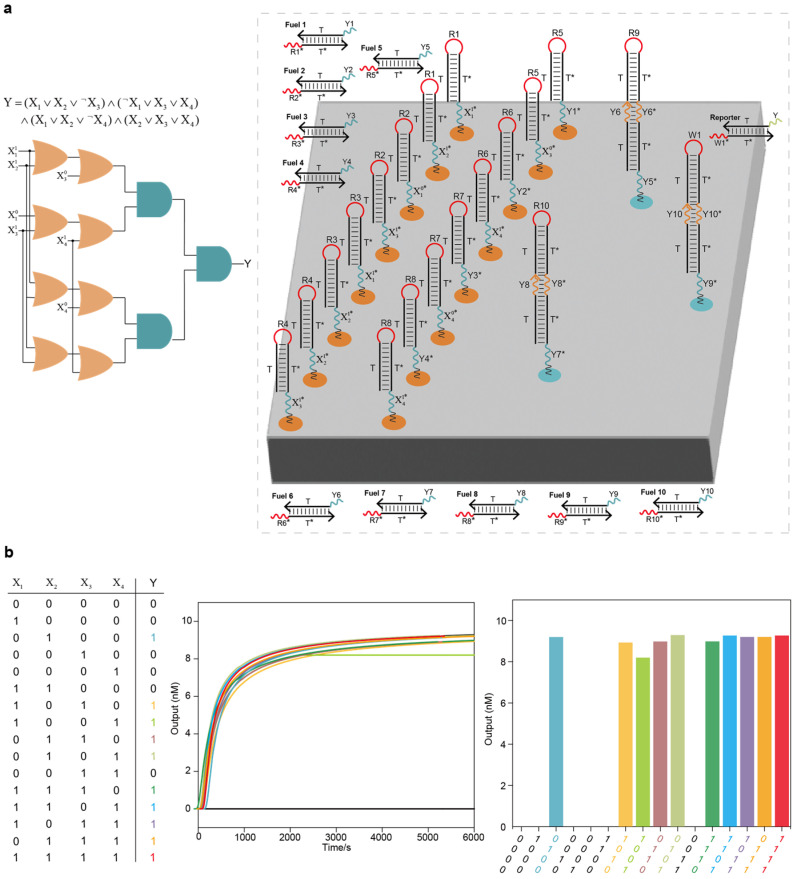
Integrated DNA logic circuit system for solving the 3-SAT problem: (**a**) The 3-SAT problem with 4 variables and the layout of the integrated DNA logic circuit system on the DNA origami surface. The entire system had 4 layers, including 8 OR circuits, 3 AND circuits, 10 fuel strands and 1 reporter molecule. (**b**) Test results under all input combinations using Visual DSD software. There were 10 solutions to this 3-SAT problem in the data pool of 16 possible solutions.

To further demonstrate the power of the constructed DNA circuits in solving complex NP (Non-deterministic Polynomial)-complete problems, we used the constructed elementary logic circuits to integrate a DNA logic circuit system for solving the 3-SAT problem with four variables (Figure 5a and Supplementary Text S1.2). The layout of the integrated DNA logic circuit system on the DNA origami surface is shown in Figure 5a. The integrated circuit system was more complex than the circuit systems of square root, full adder and full subtractor, and the entire circuit system had four layers, including 8 OR circuits, 3 AND circuits, 10 fuel strands and 1 reporter molecule. We tested all 16 possible input combinations, and the integrated DNA logic circuit system was able to accurately acquire 10 solutions to the 3-SAT problem from the data pool of 16 possible solutions (Figure 5b).

Considering that 19 localized circuit components were required to solve the 3-SAT problem with 4 clauses and that there are nearly 100 addressable sites on the DNA origami surface, we estimated that up to 60 (20 clauses, 60 variables) variables of the 3-SAT problems were allowed to be solved (Supplementary Figure S8), making it possible to solve the larger scale 3-SAT problem with the integrated DNA circuit system.

### 2.4. Localized DNA Logic Circuit Systems for Disease Classification

**Figure 6:**
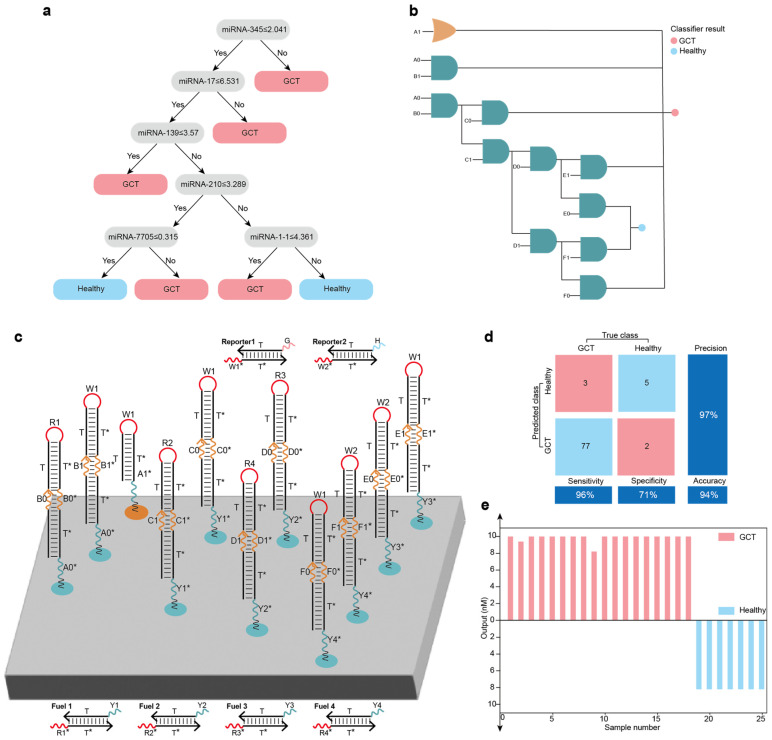
Localized DNA logic circuit systems for disease classification: (**a**) A decision tree model for GCT classification based on miRNA expression data of GCT samples from TCGA and the random forest algorithm. (**b**) The decision tree model mapping into the logic circuit system. (**c**) The layout of the localized DNA logic circuit system for GCT classification. (**d**) Confusion matrix analysis of the 87 samples. (**e**) Testing of the GCT classification for all 25 randomized samples showed agreement between the actual disease states labeled in TCGA, the random forest model and the constructed localized DNA logic circuit system.

DNA logic circuit systems have excellent analysis and decision-making capabilities. We scaled up the constructed elementary logic circuits into a localized DNA logic circuit system for germ cell tumor (GCT) state classification (healthy or diseased). A strong correlation exists between microRNA (miRNA) expression levels and cancer. We used the miRNA expression data of GCT samples from The Cancer Genome Atlas (TCGA) and the random forest algorithm to train the model (263 GCT and 23 healthy samples), and screened out the characteristic miRNAs (miRNA-345, miRNA-17, miRNA-139, miRNA-210, miRNA-7705 and miRNA-1-1) associated with GCT (Figure 6a). We used a localized

DNA logic circuit system containing 1 OR circuit, 10 AND circuits, 4 fuel strands and 2 reporter molecules to map the decision tree of miRNAs (Figure 6b,c), and then classified the state of GCT samples based on the miRNA expression levels. The miRNA expression level below the threshold was set as the logical signal X0, and above the threshold was set as the logical signal X1. We used 25 randomized miRNA expression samples from GCT samples from TCGA to test the constructed localized DNA logic circuit system for performing disease classification. Visual DSD software was used to test the analysis and classification of each dataset. The results showed agreement between the actual disease states labeled in TCGA, the random forest model and the constructed localized DNA logic circuit system (Figure 6d,e and Supplementary Figure S9).

## 3. Discussion

Herein, we demonstrated a strategy for constructing localized scalable DNA logic circuit systems based on the DNA origami surface. We first constructed elementary two-input AND and OR logic circuits on the DNA origami surface using only one and two circuit components, respectively. We then showed that the constructed elementary logic circuits can be scaled up to an arbitrary localized circuit system to implement functions such as square root circuit, full adder and full subtractor. The constructed localized scalable DNA logic circuit system based on the DNA origami surface has several advantages as follows: (1) The first is scalability, whereby the elementary two-input AND and OR logic circuits can be scaled up into large-scale localized DNA logic circuit systems. In addition, compared with the threshold strategy, our strategy involved the use of fewer circuit components on the DNA origami surface, which effectively avoids signal attenuation caused by having many components. (2) The second is modularity, which allows circuit components to be embedded anywhere in the system, and different logic functions are realized through reasonable cascading design. (3) The third is strong functionality; the constructed localized DNA logic circuit system can be effectively applied to digital computing and disease diagnosis (such as solving 3-SAT problems and classification of disease states).

We believe that the programmability and scalability of the constructed localized DNA logic circuit systems can be extended in the fields of digital computation, disease diagnosis and NP-complete problem solving. However, there are challenges that need to be addressed as the size of the problem or the amount of data associated with the disease increases. Such challenges include the following: (1) Further scaling of the size of the localized DNA logic circuit systems is constrained by the size of the DNA origami, and DNA origami of a larger size is required. (2) When performing disease diagnosis, the logic circuit system can only passively receive the 0/1 signal (for example, the miRNA expression level is set to 1 above the threshold concentration and 0 below the threshold concentration) and realize a classification decision, and logic circuit system cannot directly read the miRNA expression level above/below the threshold concentration from the samples, which impacts the efficiency and integration of disease diagnosis. Introducing a DNA chemical reaction network that can directly read the expression level of miRNA represents a potential future direction.

## 4. Materials and Methods

### 4.1. Simulation Experiments Based on Visual DSD Software

Visual DSD is a programming language and software tool for simulating DNA computing systems based on SDR. It was originally proposed by Andrew Phillips and coworkers in 2009 and has been extended with modeling, embedding biochemical experimental data and analysis capabilities [33,34,35,36,37,38,39]. Visual DSD can automatically demonstrate all possible displacement reactions between all kinds of DNA without the need to manually construct a reaction network. Visual DSD has become a powerful tool for visualizing and simulating DNA computing systems based on SDR. The Visual DSD software version used in this paper is ‘v2015-0325’. The user manual for the Visual DSD software is available online at “UPL: https://www.microsoft.com/en-us/research/uploads/prod/2009/02/Visual_DSD_Manual.pdf (accessed on 1 February 2025).

To analyze the process of SDR and predict the performance of the DNA circuits, the parameters in the Visual DSD simulation programs were set as shown in Table 1, which are able to simulate all the possible reactions between the DNA molecules.

### 4.2. Silicon-Based Training of Germ Cell Tumor (GCT) Data

We used the miRNA expression data of GCT samples from The Cancer Genome Atlas (TCGA) and the random forest algorithm to train the model (263 GCT and 23 healthy samples), with 60% of samples used for training and 40% of samples used for testing. Random forests achieve classification or regression by integrating multiple decision trees based on different sample subsets and feature subsets, and the classification results are determined by majority voting:

(1) $$\overset{\wedge }{y}=\arg\max_{c}\sum_{t=1}^{T}∐(h_{t}(X)=c)$$

or by the mean of the regression tree:

(2) $$\overset{\wedge }{y}=\frac{1}{T}\sum_{t=1}^{T}h_{t}(X)$$

The experimental environment was Windows 10 operating system and Intel Core i5. We screened out the characteristic miRNAs (miRNA-345, miRNA-17, miRNA-139, miRNA-210, miRNA-7705 and miRNA-1-1) associated with GCT, achieving 94% accuracy in GCT identification.

## 5. Conclusions

DNA origami provides a nanoscale addressable surface for the construction of DNA logic circuit systems. These localized DNA logic circuit systems constrained on the DNA origami surface have faster reaction dynamics than discrete DNA logic circuit systems. Therefore, in this paper, we proposed a strategy for constructing localized scalable DNA logic circuit systems based on a DNA origami surface and further implemented complex logic circuit systems such as square root circuit, full adder, full subtractor and logic circuit systems for solving 3-SAT problems and disease classification. This strategy involved the use of fewer circuit components on the DNA origami surface to achieve the construction of DNA logic circuit systems. We verified the feasibility, scalability and stability of the constructed localized DNA logic circuit systems through simulation experiments based on Visual DSD software. This work is expected to provide a solid foundation for intelligent integrated DNA computing systems.

## Figures and Tables

**Table 1:** The main parameters of the Visual DSD simulation programs.

Species	Parameters
*The lengths of all toeholds*	6 nt
*The lengths of all recognition domains*	20 nt
*Binding and unbinding rates*	0.003 s^−1^ for the binding rate
	0.1 s^−1^ for the unbind rate
*Directive* (defined using the directive keyword and determine how the DSD program should be executed)	Simulator deterministic
	Compilation infinite
	Polymers

## Data Availability

Data are contained within the article and Supplementary Materials.

## Supplementary Materials

The following supporting information can be downloaded at https://www.mdpi.com/article/10.3390/ijms26052043/s1. Refs. [40,41,42,43,44] are cited in the Supplementary Materials.

## Abbreviations

The following abbreviations are used in this manuscript:

SDR	Strand Displacement Reaction
3-SAT	Three-Satisfiability
miRNA	MicroRNA
GCT	Germ Cell Tumor
TCGA	The Cancer Genome Atlas
NP	Non-deterministic Polynomial
DNA	Deoxyribonucleic Acid
nt	nucleotide
