# Supplementary material for: A Localized Scalable DNA Logic Circuit System Based on the DNA Origami Surface
Source: Int J Mol Sci. 2025 Feb 26;26(5):2043. doi: 10.3390/ijms26052043 (PMC11900131; doi:10.3390/ijms26052043)
Supplement: Supplementary file 1 [file ijms-26-02043-s001.zip › ijms-3494920-supplementary.pdf]

Supplementary Materials for

A localized scalable DNA logic circuit system based on the DNA origami surface

Zhen Tang[1], Shiyin Li[1], Chunlin Chen[1], Zhaohua Zhou[1] and Zhixiang Yin[1,2,*]

1 - School of Mathematics, Physics and Statistics, Shanghai University of Engineering Science, Shanghai, 201620, China.
2 - Institute for Frontier Medical Technology, Shanghai Frontiers Science Research Center for Druggability of Cardiovascular noncoding RNA, Center of Intelligent Computing and Applied Statistics, Shanghai University of Engineering Science, Shanghai, 201620, China.

*Correspondence to Zhixiang Yin: 21190006@sues.edu.cn.

Contents

S1. Supplementary Texts Page 1 - 2

S2. Supplementary Figures Page 3-10

S1. Supplementary Texts

S1.1. A simple mathematical model of the anchoring efficiency of DNA logic components on the DNA origami surface

The two-dimensional DNA origami structure is generally folded from a long scaffold and more than 200 staples, and its surfaces has a wealth of nanoscale addressable sites, which mainly depends on the anchoring efficiency of the staples. Due to thermodynamic fluctuations, origami structure design and solution environment, the anchoring efficiency of staples varies slightly in different regions of the DNA origami surface [40,41]. Here, a simple mathematical model was developed in order to analyze and evaluate the effect of the anchoring efficiency of DNA logic components on the localized DNA logic circuit systems. We approximate that each DNA logic component would be anchored to the DNA origami surface with a uniform probability P during the annealing assembly of DNA origami. The complete execution of a localized DNA logic circuit systems requires that the DNA logic components are all successfully anchored. Accordingly, the probabilities that the localized DNA logic circuit systems under the threshold strategy and our strategy can operate completely are shown in the Table S1 and Fig. S1.

Table S1. The effect of the anchoring efficiency of DNA logic components on the localized DNA logic circuit systems

Strategy	Localized DNA logic circuit systems	Probability
Threshold strategy	elementary two-input AND	P^4
	elementary two-input OR	P^3
	OR-AND	P^6
	square root of a four-bit binary number	P^{12}
Our strategy	elementary two-input AND	P
	elementary two-input OR	P^2
	OR-AND	P^3
	square root of a four-bit binary number	P^6

S1.2. Three-satisfiability (3-SAT) problem

SAT problem is a classical NP-complete problem, which is widely studied in the field of intelligent computing such as DNA computing and artificial intelligence to serve as a benchmark for testing the performance of computational models [42,43]. A SAT problem is a problem of determining whether there is a truth-valued assignment that makes a given normal form. A normal form that usually consists of the conjunctive of several disjunctive is called a conjunctive normal form (CNF). A normal form that usually consists of the disjunctive of several conjunctive is called a disjunctive normal form (DNF). For example: $C_1 \wedge C_2 \wedge \cdots \wedge C_m$ is called a CNF, where C_i is a disjunctive. For a CNF (or DNF) with n variables, the question of whether there is a set (multiple sets) of variables whose values make the normal form true (truth value 1) is called the SAT problem. Each variable in a SAT problem takes the value of 0 or 1. The mathematical expression of a SAT problem is as follows:

$$F = \mathop{\wedge}_{i=1}^{m} C_i = \mathop{\wedge}_{i=1}^{m} \mathop{\vee}_{j=1}^{m} L_{i,j}$$

If all clauses of a CNF (or DNF) contain K variables, the SAT problem is called K-SAT. The 3-SAT problem is K=3, that is, each clause contains 3 variables. The mathematical expression of the 3-SAT problem we used is as follows:

$$F = (X_1 \vee X_2 \vee \neg X_3) \wedge (\neg X_1 \vee X_3 \vee X_4) \wedge (X_1 \vee X_2 \vee \neg X_4) \wedge (X_2 \vee X_3 \vee X_4)$$

S2. Supplementary Figures

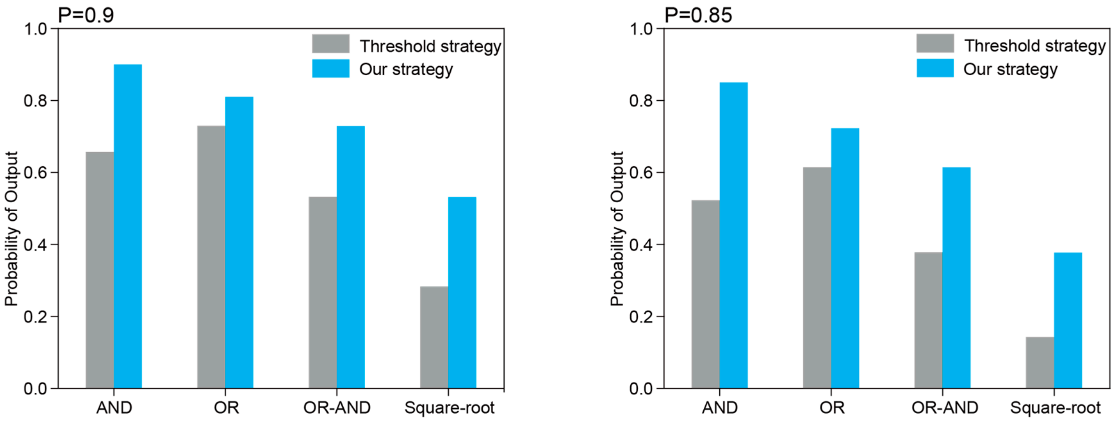

Figure S1. The effect of the anchoring efficiency of DNA logic components on the localized DNA logic circuit systems. Due to thermodynamic fluctuations, origami structure design and solution environment, the anchoring efficiency of staples varies slightly in different regions of the DNA origami surface [40,41]. We approximate that each DNA logic component would be anchored to the DNA origami surface with a uniform probability P during the annealing assembly of DNA origami. Here, we compared the effects of our strategy and the threshold strategy on the localized DNA logic circuit systems under P=0.9 and P=0.85 conditions. With the increase the complexity of the localized DNA logic circuit systems, our strategy can better maintain the integrity of circuit performance.

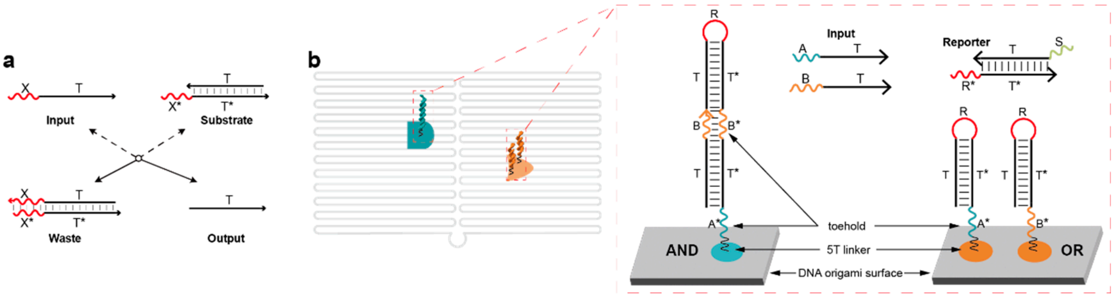

Figure S2. Strand displacement reaction (SDR) and elementary DNA logic circuits on the DNA origami surface. **a.** The principle of SDR. A single-stranded DNA <X T> acting as the input signal binds to the complex substrate via a toehold X, and displaces the signal strand <T> in an entropy-driven manner. **b.** The elementary DNA logic circuits consisted of the circuit components anchored to the DNA origami surface and the reporter molecules. The design of the DNA origami was derived from Ref. [44]. The circuit components were confined to the origami surface by extending the poly 5T linker through staples within the DNA origami.

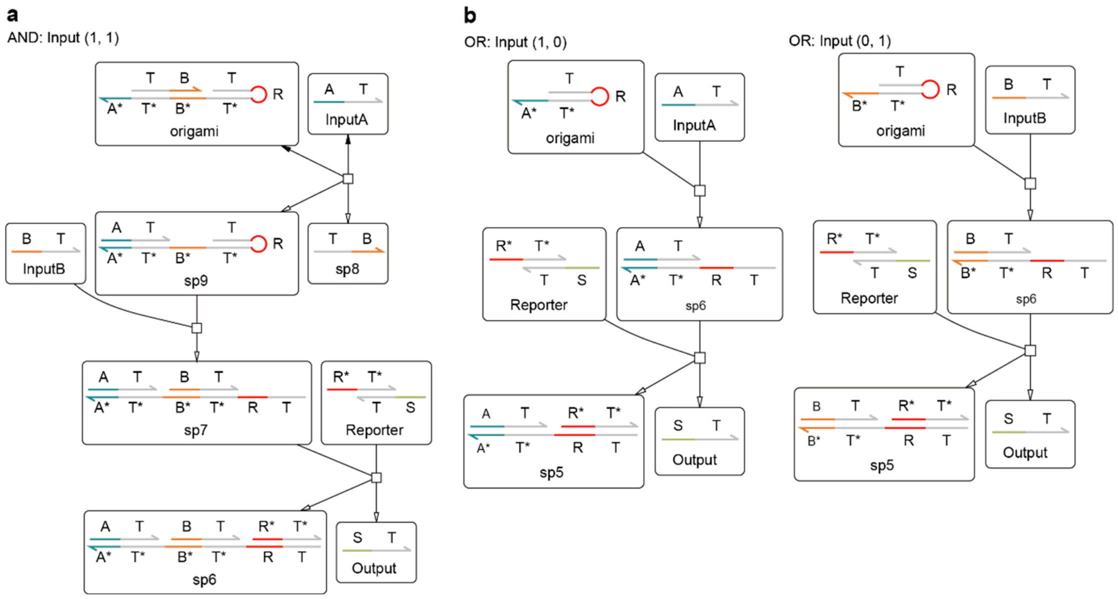

Figure S3. Visualize the reaction details of the logic operations of the elementary two-input logic circuits from Visual DSD software. **a.** The elementary two-input AND logic circuit. When the two input strands were added, signal strand A first bound to toehold A*, and toehold B* was exposed with SDR. Signal strand B bound to toehold B*, the hairpin of the DNA complex was turned on, and the exposed toehold R bound to the toehold R* of the reporter molecule, resulting in a high output signal. **b.** The elementary two-input AND logic circuit. Once an input strand was added, the hairpin of the DNA strand was opened, and the exposed toehold R bound to the toehold R* of the reporter molecule, resulting in a high output signal.

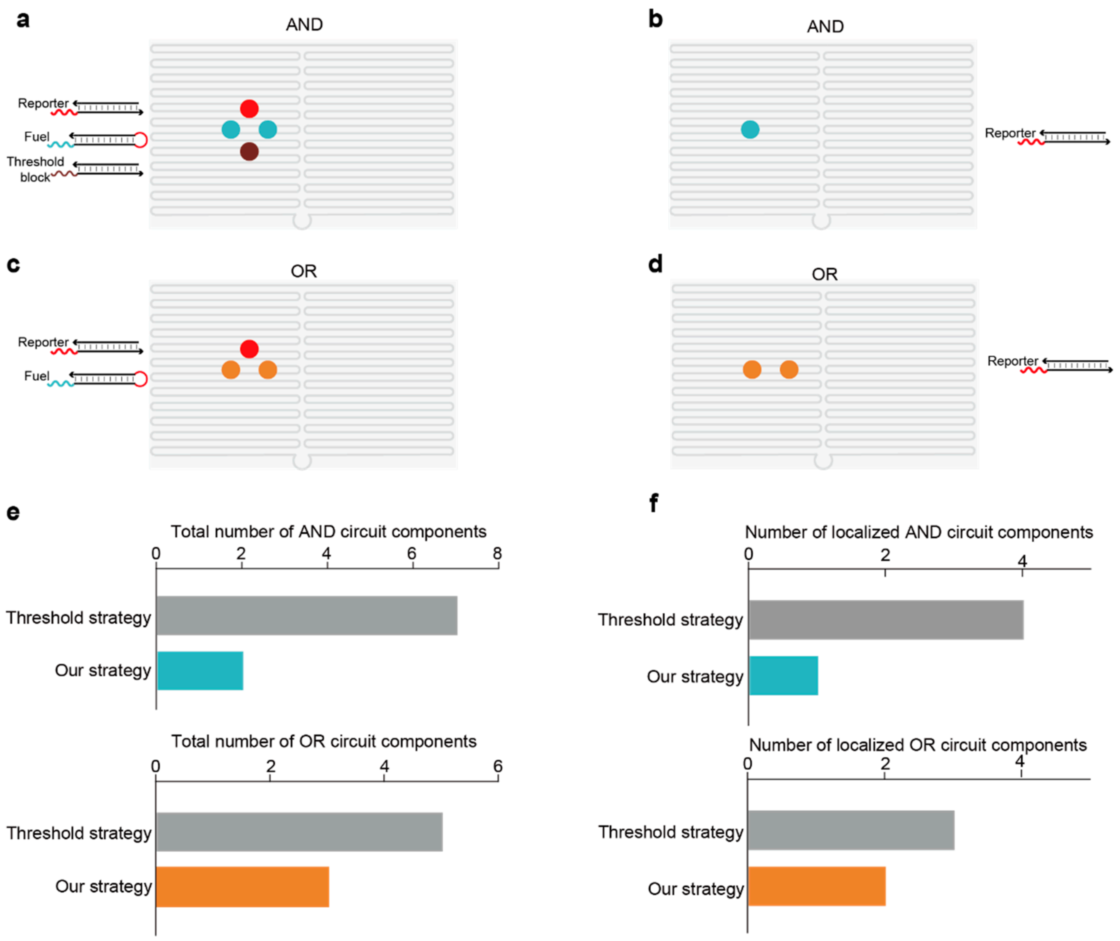

Figure S4. Comparison of the elementary localized DNA logic circuit systems based on threshold strategy and our strategy. **a.** The elementary two-input AND logic circuit system based on threshold strategy. The entire system had 7 circuit components, 4 of which were anchored on the DNA origami surface. **b.** The elementary two-input AND logic circuit system based on our strategy. The entire system had 2 circuit components, 1 of which were anchored on the DNA origami surface. **c.** The elementary two-input OR logic circuit system based on threshold strategy. The entire system had 5 circuit components, 3 of which were anchored on the DNA origami surface. **d.** The elementary two-input OR logic circuit system based on our strategy. The entire system had 3 circuit components, 2 of which were anchored on the DNA origami surface. **e. f.** Compared to the threshold strategy, the number of circuit components for the elementary two-input AND logic circuit we constructed was reduced by nearly 71%, with the number of circuit components on DNA origami surface reduced by nearly 75%. The number of circuit components for the elementary two-input OR logic circuit we constructed was reduced by nearly 40%, with the number of circuit components on DNA origami surface reduced by nearly 33%.

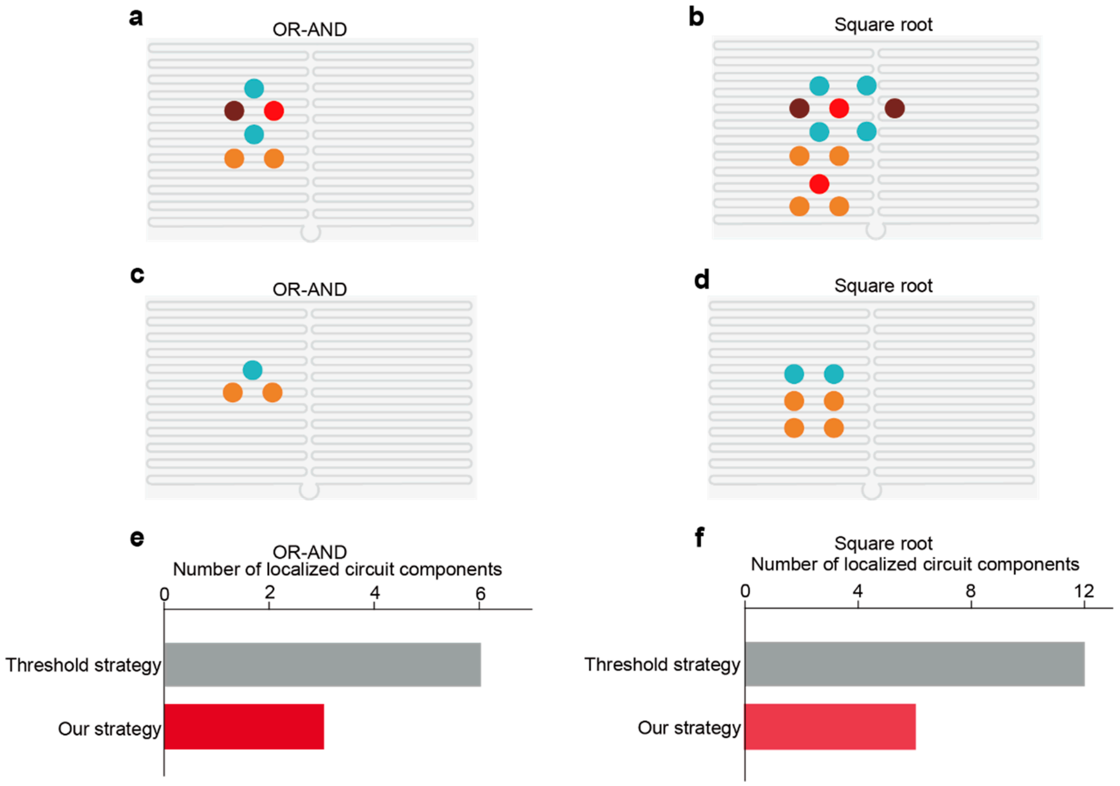

Figure S5. Comparison of the localized OR-AND and square root logic circuit systems based on threshold strategy and our strategy. **a.** The localized OR-AND logic circuit system based on threshold strategy. The entire system had 6 DNA components on the DNA origami surface. **b.** The localized square root logic circuit system based on threshold strategy. The entire system had 12 DNA components on the DNA origami surface. **c.** The localized OR-AND logic circuit system based on our strategy. The entire system had 3 DNA components on the DNA origami surface. **d.** The localized square root logic circuit system based on our strategy. The entire system had 6 DNA components on the DNA origami surface. **e. f.** Compared to the threshold strategy, the number of localized circuit components for the OR-AND and square root logic circuit systems we constructed were reduced by nearly 50%, respectively.

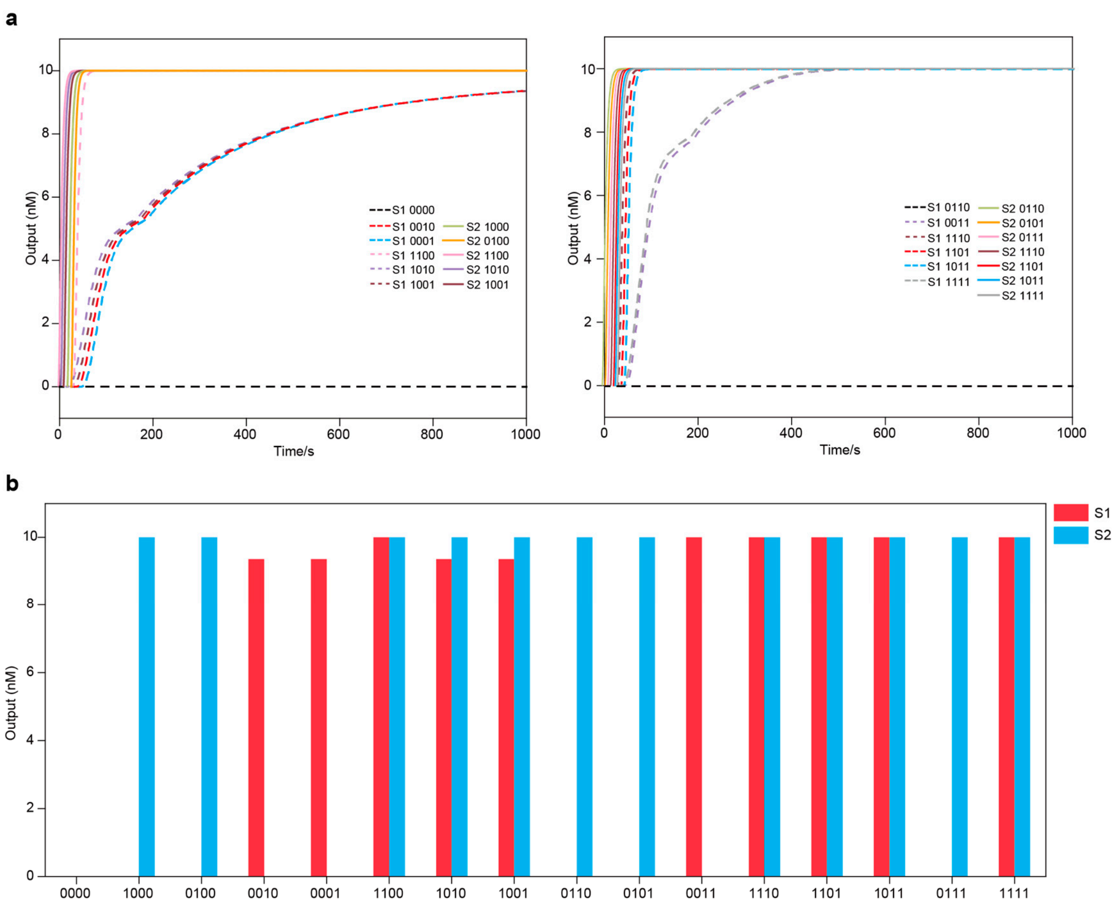

Figure S6. Test results of the localized DNA logic circuit system for computing the floor of the square root of a four-bit binary number under all input combinations using Visual DSD software. All the outputs went to the correct signals.

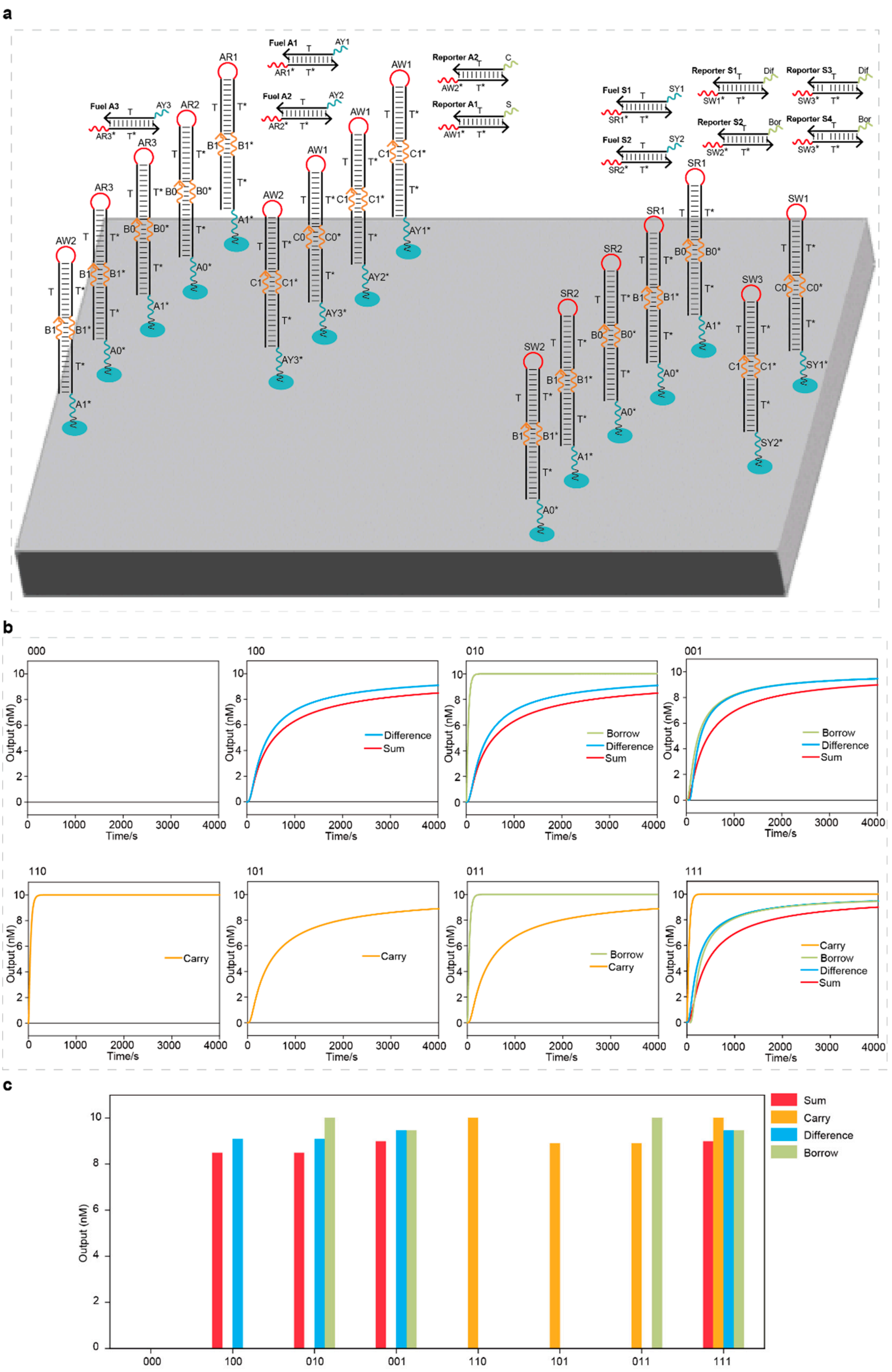

Figure S7. Parallel computing of DNA full adder and full subtractor circuit systems on the DNA origami surface. **a.** The layout of the full adder and full subtractor circuit systems on the same DNA origami surface. **b. c.** Test results of parallel computing of full adder and full subtractor circuit systems using Visual DSD software. All the outputs went to the

correct signals.

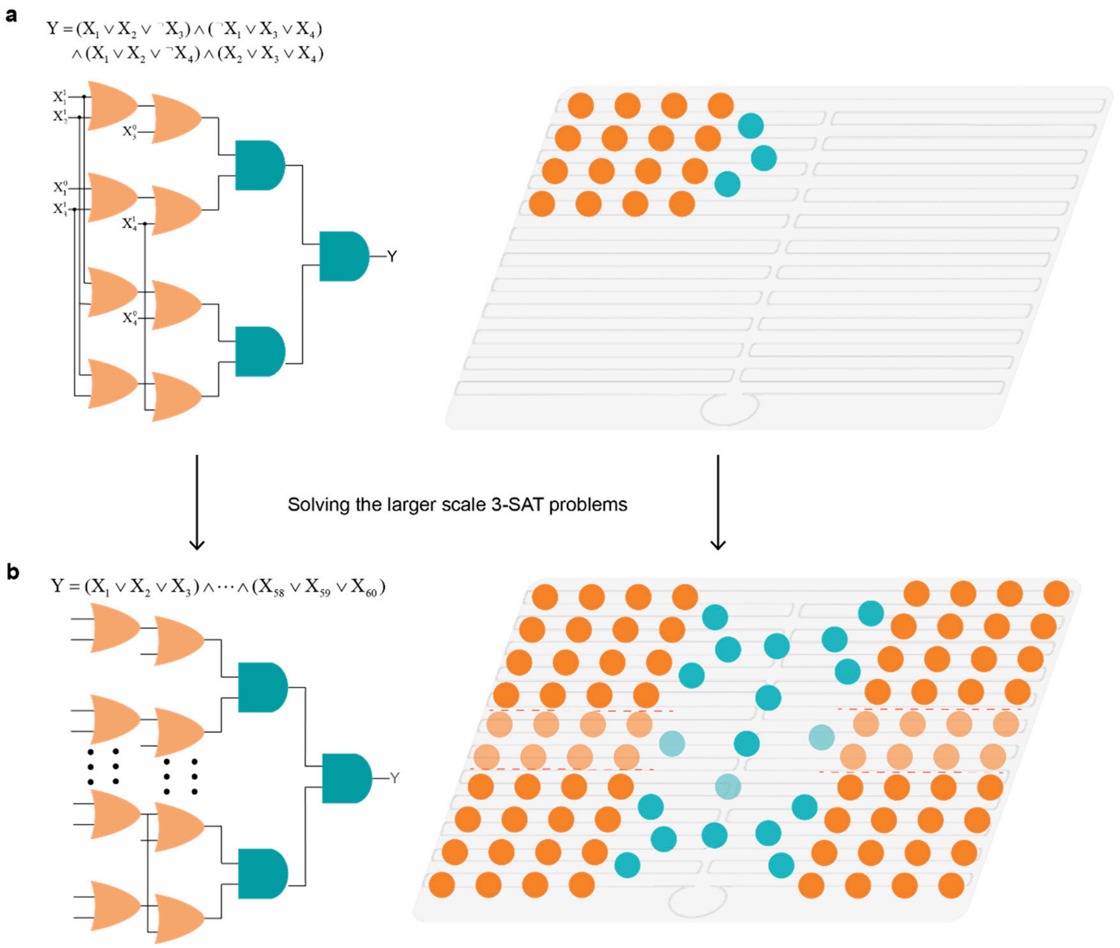

Figure S8. The localized DNA logic circuit system for the larger 3-SAT problem. **a.** For the 3-SAT problem with 4 variables, the circuit system had 19 localized circuit components, including 8 OR circuits, 3 AND circuits. **b.** Considering that there are nearly 100 addressable sites on the DNA origami surface, we estimated that up to 60 (20 clauses, 60 variables) variables of the 3-SAT problems were allowed to be solved.

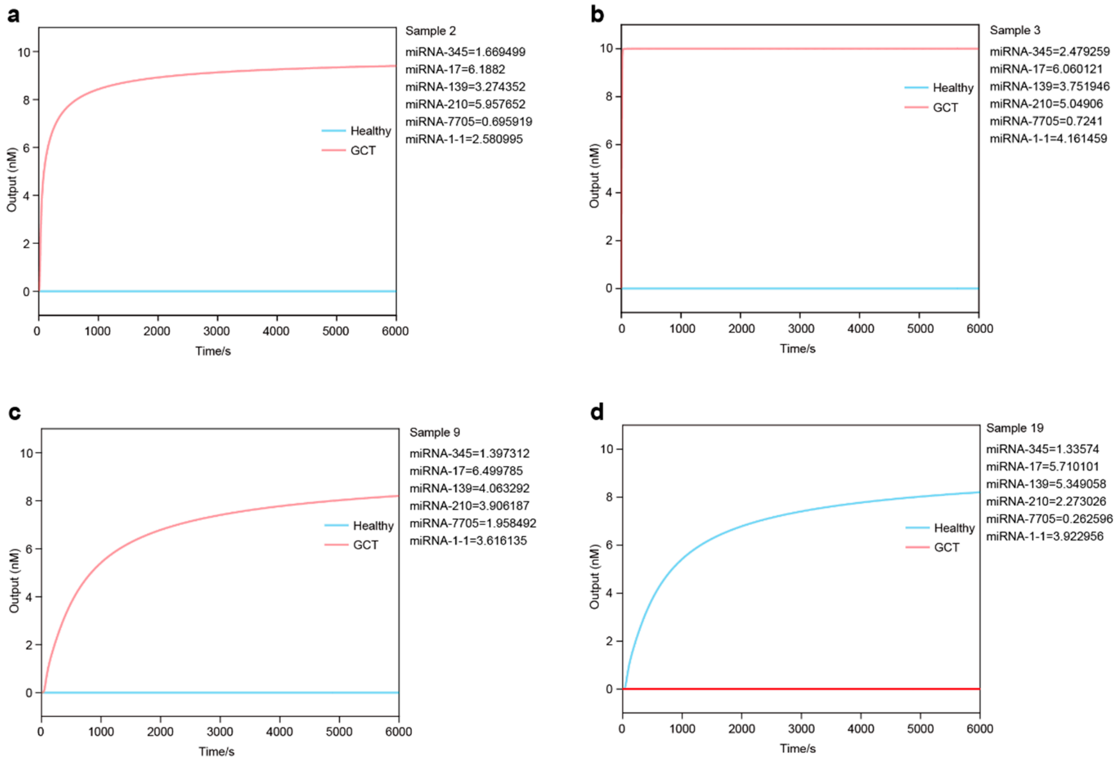

Figure S9. Four representative miRNA expression samples from the TCGA of GCT were tested by the constructed localized disease classification DNA logic circuit system.
